# Performance and workflow comparison of the VITEK MS PRIME and Bruker Biotyper MALDI-TOF MS systems

**DOI:** 10.1128/jcm.00211-25

**Published:** 2025-06-20

**Authors:** Rachel E. Bosserman, Nicole J. Tarlton, Kelly Alvarado, Brittany Roemmich, Melanie L. Yarbrough

**Affiliations:** 1 Department of Pathology and Immunology, Washington University School of Medicine 12275, St. Louis, Missouri, USA; Johns Hopkins University, Baltimore, Maryland, USA

**Keywords:** MALDI-TOF MS, VITEK MS PRIME, MALDI Biotyper

## Abstract

Matrix-assisted laser desorption ionization time-of-flight mass spectrometry (MALDI-TOF MS) is widely used for rapid identification of bacteria and yeast in clinical labs. The VITEK MS PRIME (“PRIME”) is the latest MALDI-TOF MS system from bioMérieux. This study evaluated PRIME’s performance and workflow timing against the MALDI Biotyper CA System by Bruker (“Biotyper”). We compared a collection of 154 bacteria and yeast clinical isolates from various specimen types using three methods: Biotyper target with a toothpick, PRIME target with the PICKME nib, and PRIME target with a loop. Positive blood culture isolates were also analyzed using these methods, with high-experience (HEU, *n* = 300) and low-experience users (LEU, *n* = 50) after short (6–8 h) and routine (18–24 h) incubation on agar plates. Workflow timing, from sample processing to organism identification, was assessed to identify time savings. PRIME identified 96% (PICKME) and 95% (Loop) of challenge isolates to genus level, compared with Biotyper at 99%. Short incubation of positive blood culture isolates demonstrated similar species-level identification rates across all methods (Biotyper: 84%, PRIME PICKME: 80%, PRIME Loop: 81%), although more repeats were needed compared with routine incubation. No difference in species identification occurred between users for any method at short incubation (89%–91%, HEU, vs 79%–85% LEU). Single-target process times were comparable for all methods (55–59 min), whereas PRIME methods had shorter hands-on times for analysis of multiple targets (Biotyper: 53 min, PRIME PICKME: 39 min, PRIME Loop: 40 min). These findings highlight the comparable performance of the PRIME and Biotyper systems while demonstrating the potential for time savings with PRIME workflows, particularly in high-throughput settings.

**IMPORTANCE:**

This study provides a critical evaluation of the new VITEK MS PRIME MALDI-TOF MS system, comparing its performance and workflow efficiency against the Bruker MALDI Biotyper. The study investigates the success rate of isolate identification from short incubation of positive blood cultures, illustrating the utility of this technique for downstream workflows such as faster reporting of results for patient management and isolate identification for interpretation of rapid phenotypic AST. Analysis of different workflows demonstrated areas for potential time savings, particularly in high-throughput settings. These findings highlight the importance of optimizing MALDI-TOF MS workflows in the era of workforce shortages and lab centralization to enhance rapid pathogen identification and improve patient care.

## INTRODUCTION

Matrix-assisted laser desorption ionization time-of-flight mass spectrometry (MALDI-TOF MS) has become an integral tool in contemporary clinical microbiology laboratories, facilitating the accurate and reliable identification of bacteria and yeast (1–5). FDA-cleared MALDI-TOF MS instruments are commercially available from Bruker and bioMérieux. The latest addition to the bioMérieux MALDI-TOF MS instrument portfolio is the VITEK MS PRIME ("PRIME"), which introduces several enhancements over its predecessor that may lead to improved workflows in clinical microbiology laboratories (6).

Notable features of the PRIME system include a continuous load-and-go sample loading mechanism, an "urgent" analysis option, and a reduction in slide analysis time. One of the primary distinctions between the PRIME and the Bruker MALDI Biotyper ("Biotyper") systems lies in the process of target setup and loading. The PRIME allows for the loading of up to 16 target slides simultaneously, with the flexibility to load additional targets while the acquisition of another target is underway. In contrast, the Biotyper loads and processes only one target at a time. The PRIME utilizes targets with three acquisition groups, each comprising 16 spots. If an acquisition group is not fully utilized, any unused spots in that group cannot be used in subsequent analyses. Conversely, the Biotyper employs target plates with 48 or 96 spots, depending on whether the target is disposable or reusable. Unused spots on Biotyper targets can be used in additional analyses until all spots have been utilized. A detailed comparison of PRIME and Biotyper features is presented in Table S1.

The rapid identification of microorganisms causing bloodstream infections is crucial for initiating timely and targeted antimicrobial therapy (7). Traditional identification workflows rely on the 18–24 h growth of isolated bacterial colonies. Identification methods using MALDI-TOF MS have demonstrated the ability to rapidly identify microorganisms directly from positive blood cultures (8, 9). These methods require several hands-on steps to concentrate microorganisms and eliminate interfering proteins before analysis. This additional labor, time, cost, and modification to existing workflows may not be feasible in all laboratory settings. MALDI-TOF MS has been shown to successfully identify bacteria from early growth cultures without the need for extraction or additional processing steps (10, 11), which would allow same-day identification in many cases. To this end, we evaluated the identification of early growth from positive blood culture broths using both Biotyper and PRIME. Additionally, we compared the timing of steps, from sample processing to organism identification for various workflows, on Biotyper and PRIME to determine whether either system offered a notable time-savings advantage for specific workflow types.

## MATERIALS AND METHODS

This study was conducted at a large academic medical center in St. Louis, Missouri, USA, and approved by the Washington University School of Medicine Human Research Protection Office (IRB #202205052). Isolates from human clinical specimens were enrolled under a waiver of consent.

### Isolate preparation for performance evaluation and workflow analysis

Isolates were collected in real time or previously frozen from patient specimens at Barnes-Jewish Hospital (BJH) in St. Louis, MO. Isolates were from a variety of culture types collected for routine patient care, including bone, tissue, sterile body fluids, wounds, abscesses, respiratory, hardware, and blood cultures. This challenge set was selected to ensure a broad array of organisms were tested on the PRIME. Isolates were subcultured (twice for frozen isolates) to blood agar plates (Hardy Diagnostics) for bacteria or Sabouraud dextrose with chloramphenicol agar plates (Hardy Diagnostics) for yeast and incubated for 18–24 h at 35°C in 5% CO_2_. Anaerobic bacteria were subcultured to Brucella blood agar plates (Hardy Diagnostics) and incubated anaerobically for 48 h at 35°C.

### Target preparation and MALDI-TOF MS identification

#### Biotyper identification method (standard of care [SOC])

Isolates were spotted with a toothpick onto a Biotyper steel target and overlayed with 1 µL of formic acid, allowed to dry, and then overlayed with 1 µL of matrix (α-Cyano-4-hydroxycinnamic acid [CHCA], reconstituted in Bruker standard solvent) and allowed to dry, according to standard operating procedures for the BJH clinical laboratory. MALDI-TOF MS spectra were collected by the instrument and analyzed using the MBT-BDAL-10833 Bruker database. Acceptable species-level identifications were determined per the standard of care protocol validated by our laboratory. Briefly, yeasts were interpreted to the species level at a score ≥1.7, gram-positive cocci were interpreted to the species level at a score ≥1.8, and a score of ≥2.0 was required for species-level identification of gram-negative bacteria, except Enterobacterales and *Bacteroides fragilis*, which required a score of 1.8. Scores of ≥1.7 were acceptable for a genus-level identification.

#### PRIME identification methods

Isolates were spotted onto a PRIME target using two different methods—the PICKME nib (PRIME PICKME) or a plastic loop (PRIME Loop). Colonies were overlaid with 1 µL of the matrix alone for bacteria, or 1 µL of formic acid then 1 µL of the matrix (VITEK MS-CHCA) for yeast. MALDI-TOF MS spectra were collected and analyzed according to the KB v3.2 VITEK MS PRIME database. Identification level was reported by the instrument software; a “good” identification was considered a species-level identification, whereas a “low discrimination” with split identifications was considered a genus-level identification. No further testing was done for unclaimed organisms unless the identification was discrepant between other methods.

#### Repeat analysis for no identification

For the PRIME methods, if no identification was obtained the isolate was re-spotted, and MALDI-TOF MS identification was attempted a second time. Organisms with results of no identification by the PRIME were investigated further by sequencing, as described below. For the Biotyper, there were 2 scenarios on the report: (i) no peaks found and (ii) no identification (i.e., score <1.7). If no peaks were found, the isolate was re-spotted, and MALDI-TOF MS identification was attempted a second time. If there was no identification, the original isolate spot was reanalyzed manually (240 spectra collected, 40% laser intensity) in the second attempt. For all methods, if the second attempt resulted in no identification, the result was considered no identification.

### Sequencing and bioinformatic analysis of microorganisms that yielded discrepant MS results

#### Discrepant MS results

Isolate identifications were considered discrepant if identification was achieved on only one instrument or if species-level identification differed using any method. Differences in call level (i.e., genus vs species-level identification) or species-level identification of organisms routinely reported as a complex or at the genus level by our laboratory (e.g., *Salmonella* species, *Enterobacter cloacae* complex) were not considered discrepant. Discrepant isolates were further tested by whole genome sequencing.

#### DNA isolation and whole genome sequencing

Sequencing was performed by the Research and Development Microbiology Sequencing Team at bioMérieux Inc. (St. Louis, MO). Briefly, all strains were grown to logarithmic phase in a liquid growth medium and pelleted to prepare for DNA isolation. DNA was isolated using the DNeasy UltraClean Microbial Kit (Qiagen) following the manufacturer’s protocol. The DNA concentration was quantified using a Qubit 4 fluorometer (ThermoFisher Scientific) following the Qubit dsDNA HS Assay Kit protocol. DNA quality checks were performed on at least 10% of total samples using a 4150 TapeStation System (Agilent) with the D1000 ScreenTape. Genomic DNA libraries were prepared according to the Illumina DNA Prep Reference Guide (Document 1000000025416 v09), using 100 ng of DNA from each sample. Each library was normalized to 10 nM and pooled together in equimolar amounts for sequencing. Finally, 4 nM of pooled sample was denatured and loaded at 13.5 pM into a MiSeq Reagent Kit v2 cartridge for 2 × 150 paired-end sequencing on a MiSeq instrument (Illumina). The raw sequence (fastq) data were deposited in the Sequence Read Archive (SRA) of the National Center for Biotechnology Information (NCBI) under BioProject accession number PRJNA1222830 (https://www.ncbi.nlm.nih.gov/bioproject/).

#### Whole genome sequence assembly and quality assessment

Raw sequence reads were analyzed for quality and contamination by FastQC (v0.11.9) and Kraken (v2.1.2). Bacterial genomes were assembled using the Comprehensive Genome Analysis service at the Bacterial and Viral Bioinformatics Resource Center (12), which trimmed reads with Trim Galore (v0.6.5dev)/Cutadapt (v4.2) and assembled contiguous sequences with Unicycler (v0.4.8). To correct minor misassemblies, trimmed reads were mapped back to the assemblies using Samtools (v1.17)/Minimap2 (v2.17), and corrections were made using Pilon (v1.23). Fungal genomes were assembled on a Linux-based system using BBMap (v38.90), SKESA (v.2.4), Samtools (v1.7)/BWA (v0.7.17), and Pilon (v1.24). The quality of all microorganism sequence assemblies was evaluated using QUAST 5.2.0.

#### Whole genome sequence-based microorganism identification

Genome assemblies were uploaded to JSpecies WS for identification determination (13). Submitted bacterial genomes underwent a Tetra Correlation Search (TCS) analysis, which provides a list of closely related reference genomes in the JSpecies database. In some cases, genomes were submitted to the Type Strain Genome Server to determine the most closely related prokaryotic species (14). TCS or TYGS results were used to select type strain genomes for an ANIb (Average Nucleotide Identity by BLAST) analysis. An ANIb score >95% for a pair-wise comparison between a query and reference genome was considered the same species. For identification determination to the subspecies level, all subspecies-type strains with available genome sequences were included in the ANIb analysis. The genome with the highest ANIb score above 95% in comparison to the query genome was considered the subspecies identifiication. Fungal species-level IDs were determined by ANIb as described above. Reference genomes were uploaded to JSpeciesWS from GenBank.

### Identification of isolates from positive blood cultures after short and routine plate incubation

**Fig 1:**
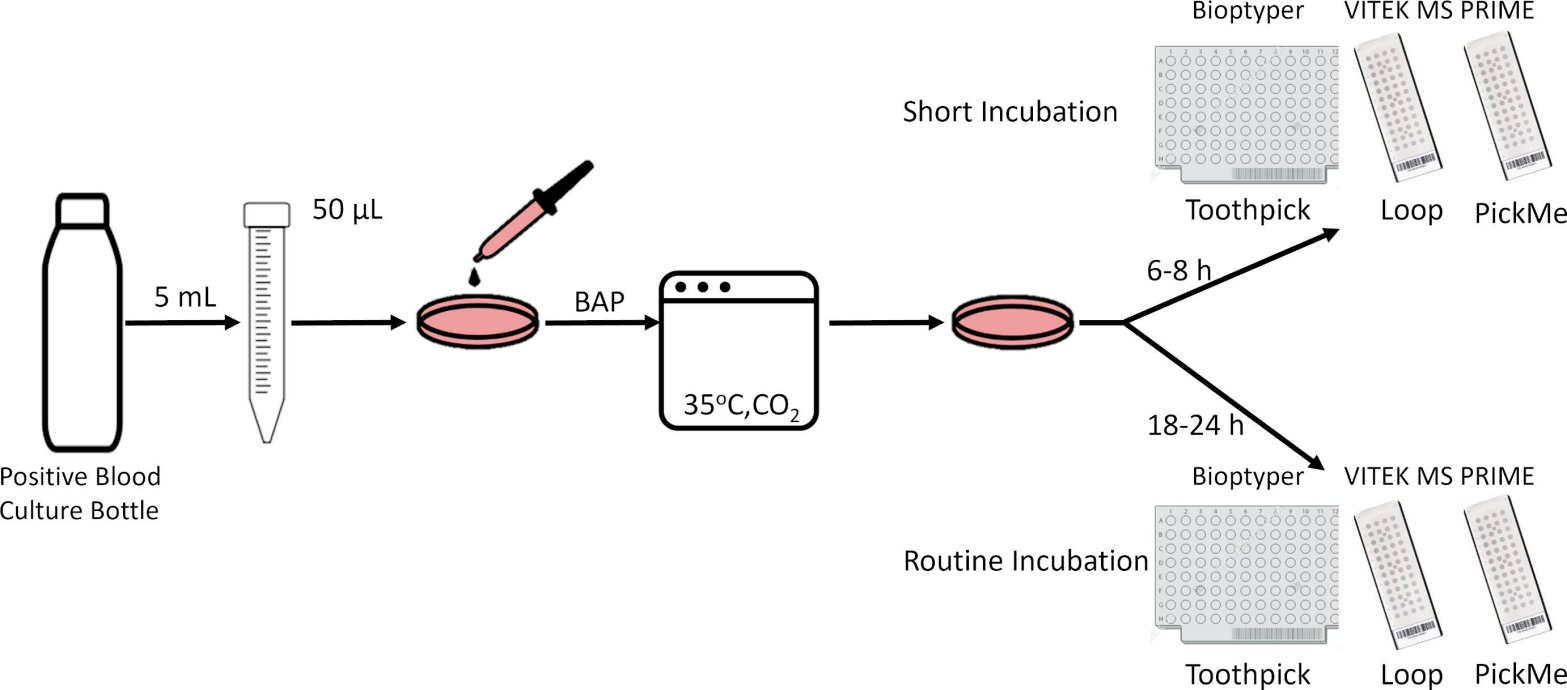
Blood culture study workflow. Positive blood cultures were plated on blood agar plates and incubated at 35°C in 5% CO_2_. Colony growth was spotted on MALDI-TOF MS targets after short (6–8 h) and routine (18–24 h) incubation periods.

Remnant positive blood culture broths from BACT/ALERT FA Plus and FN Plus bottles incubated on the bioMérieux VIRTUO instrument were obtained from the BJH Clinical Microbiology Laboratory. Positive blood culture broths were included in the study if the time to positivity was <40 h, and the bottle had signaled positive within the past 8 h. Bottles were excluded if the sample was from a subject with a positive blood culture previously enrolled in the study. As shown in Fig. 1, positive blood culture broths were plated to blood agar (when bacteria were seen on gram stain) plus Sabouraud dextrose agar plates (when yeast were seen on gram stain) and incubated at 35°C in CO_2_ for 6–8 h (short incubation) and 18–24 h (routine incubation). Isolates from 300 samples were identified by MALDI-TOF MS as described above (Table S2). For identification attempts at the short incubation stage, individual colonies (if present) or scum growth was spotted on the target (Fig. S1). Isolate identification was attempted on the Biotyper and PRIME after short and routine incubation by a high-experience user (HEU; >1 year of MALDI-TOF MS experience). A subset of specimens (*n* = 50) was also tested by a low-experience user (LEU; <1 month of MALDI-TOF MS experience). Time-to-result (TTR) was defined as the number of hours from the time of setup to the time of the MALDI-TOF MS result.

### MALDI-TOF MS laboratory workflows and timing analyses

#### Single-target workflow

**Fig 2:**
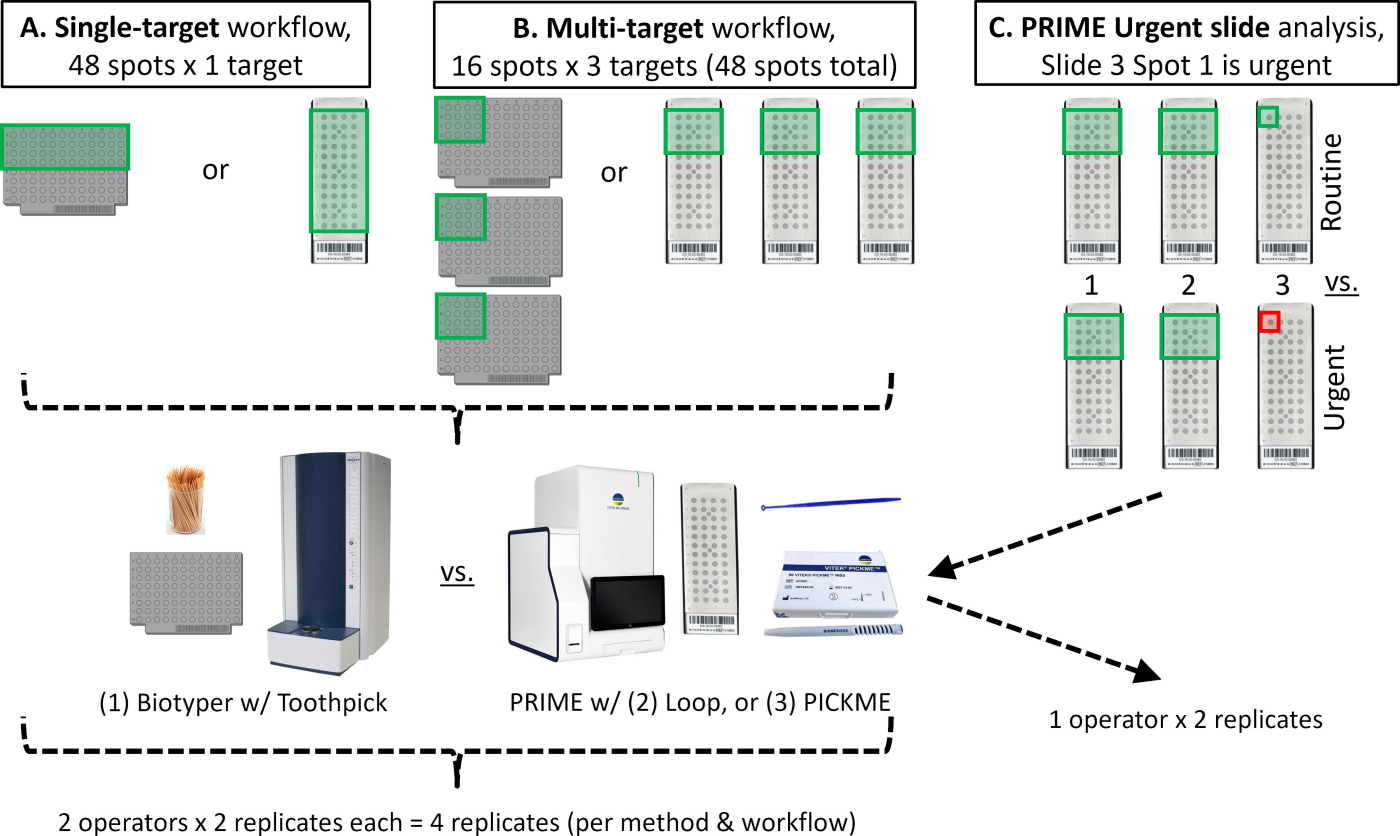
MALDI-TOF MS workflow evaluation and urgent slide analysis experiment diagrams. (**A**) Single-target and (**B**) multi-target workflow timing studies were compared for 3 methods: (i) Biotyper, (ii) PRIME loop, and (iii) PRIME PICKME. Each workflow method was performed by two operators—a high-experience user (HEU) and a low-experience user (LEU)—in duplicate. (**C**) PRIME urgent slide analyses were performed by an HEU using PICKME in duplicate, where the difference in time to identify the organism on slide 3/spot 1 was compared with and without the use of urgent analysis.

Forty-eight isolates of bacteria and yeast (four strains each of 12 organisms; Table S3 & Fig. 2) were spotted onto a single target for each method (Biotyper, PRIME PICKME, PRIME Loop). Both an HEU and an LEU performed these experiments in duplicate, for a total of four targets (four replicates) per method. Experiments were performed over 3 days to account for day-to-day instrument variability. The following steps were measured: (i) “target set-up” included the time it takes to add barcodes to a target sheet to label spots, spot the 48 isolates on the target, and add and dry formic acid (when applicable) and matrix; (ii) “instrument set-up/loading” included the time it takes for instrument setup, sample layout setup via barcode scanning, and target loading onto the instrument; and (iii) “acquire spectra/ID” included the time it takes for the vacuum seal to establish after the target is inserted, calibration, data acquisition, display of organism identifications, and result review. “Total process completion time” was defined as steps (i) + (ii) + (iii) + target unloading time. “Hands-on time” included any step where the user was actively engaged and included steps (i) and (ii) [+calibration step for Biotyper only], result review, and target unloading.

#### Multi-target workflow

The same 48 isolates (Table S3 & Fig. 2) were evenly distributed across three individual targets for each method (Biotyper, PRIME PICKME, PRIME Loop), that is, 16 spots/target x3 targets per method. Both an HEU and an LEU performed experiments in duplicate, for a total of 12 targets (four replicates) per method. Experiments were performed over 4 days to account for day-to-day instrument variability. The components measured for steps (i), (ii), (iii), “total process completion time” and “hands-on time” were the same as for the single-target workflow. However, since the multi-target workflow could be accomplished via two main pathways depending on a laboratory’s staffing and culture volume, step (i) (Target Set-up) was simulated in two different ways. The multi-tech workflow was timed to simulate three technologists working at different laboratory benches, each setting up a separate target with 16 spots (e.g., one full acquisition group) at the same time. In the one-tech workflow, simulating a laboratory with a dedicated MALDI technologist, one technologist sequentially set up three targets, each with 16 spots. “Total process completion time” was again defined as steps (i) + (ii) + (iii) + target unloading.

#### PRIME urgent slide analysis

The time savings for an organism identification when a slide is designated as “urgent” on the PRIME was evaluated by comparing the time to identification of a single *S. aureus* spot on target three of three in a “routine” vs “urgent” workflow. The three-target setup consisted of: targets 1 and 2 containing 1 full acquisition group each, with *S. aureus* as spot 1 and *E. coli* as spots 2–16, and target 3 containing only *S. aureus* in spot 1. For the routine workflow, targets 1, 2, and 3 were loaded sequentially; for the urgent workflow, targets 1, 2, and 3 were loaded sequentially, but target 3 was designated “urgent” after target 1 began processing. The time to identification timing window began at the start of the acquisition of slide 1 and ended when an organism identification for spot 1 on slide 3 was visible on the instrument. Time savings were calculated as: time to identification for “routine” minus “urgent.” An HEU performed this experiment in duplicate over 2 days to account for day-to-day instrument variability.

### Statistical analysis

Statistical analyses were conducted using GraphPad Prism 9, with significance set at *P* < 0.05. For categorical data, McNemar’s test was applied for paired groups, and Fisher’s exact test was used for unpaired data. One-way ANOVA followed by Tukey’s post hoc test was applied for comparison of numerical data. Kappa statistics were computed using R (version 4.4.1) with an empirical bootstrap resampling approach based on 1,000 resamples to estimate 95% confidence intervals and *P*-values. Multiple hypothesis testing was corrected using the Bonferroni-Holm method.

## RESULTS

### Performance evaluation of the VITEK MS PRIME using two methods to spot targets

We evaluated a diverse collection of 154 clinical isolates of either bacteria (*n* = 139) or yeast (*n* = 15) using the PRIME PICKME and PRIME Loop methods. Most (102, 66.2%) were evaluated as fresh isolates that had never been frozen, whereas 52 of the isolates were previously frozen. Sixty-six gram-positive bacteria, 60 gram-negative bacteria, 13 anaerobic bacteria, and 15 yeast identified by our SOC method (Biotyper) were included in the study. A complete list of isolates can be found in Table 1, along with the level of identification obtained by the PRIME (i.e., genus, species, or no identification). Isolates that failed to initially identify to genus- or species-level were retested one time.

**TABLE 1:** Performance evaluation of VITEK MS PRIME when spotting isolates onto a target with PICKME vs a loop

Organism	#	Genus-level ID				Species-level ID				No ID			
		PICKME		LOOP		PICKME		LOOP		PICKME		LOOP	
		No.	(%)	No.	(%)	No.	(%)	No.	(%)	No.	(%)	No.	(%)
**Gram-positive bacteria**													
*Staphylococcus aureus*	6	6	(100)	6	(100)	6	(100)	6	(100)	0	(0)	0	(0)
*Staphylococcus argenteus^a^*	5	5	(100)	5	(100)	0	(0)	0	(0)	0	(0)	0	(0)
*Staphylococcus epidermidis*	3	3	(100)	3	(100)	3	(100)	3	(100)	0	(0)	0	(0)
*Staphylococcus lugdunensis*	3	3	(100)	3	(100)	3	(100)	3	(100)	0	(0)	0	(0)
*Staphylococcus pettenkoferi*	1	0	(0)	0	(0)	0	(0)	0	(0)	1	(100)	1	(100)
*Staphylococcus pseudintermedius*	7	7	(100)	7	(100)	0	(0)	0	(0)	0	(0)	0	(0)
*Staphylococcus ureilyticus^b^*	1	1	(100)	1	(100)	1	(100)	1	(100)	0	(0)	0	(0)
*Staphylococcus warneri*	1	1	(100)	1	(100)	1	(100)	1	(100)	0	(0)	0	(0)
*Streptococcus agalactiae*	7	7	(100)	7	(100)	7	(100)	7	(100)	0	(0)	0	(0)
*Streptococcus anginosus*	2	2	(100)	2	(100)	2	(100)	2	(100)	0	(0)	0	(0)
*Streptococcus pneumoniae*	2	2	(100)	2	(100)	2	(100)	2	(100)	0	(0)	0	(0)
*Streptococcus pyogenes*	6	6	(100)	6	(100)	6	(100)	6	(100)	0	(0)	0	(0)
*Streptococcus dysgalactiae*	1	1	(100)	1	(100)	1	(100)	1	(100)	0	(0)	0	(0)
*Streptococcus minor*	1	0	(0)	0	(0)	0	(0)	0	(0)	1	(100)	1	(100)
*Enterococcus faecalis*	4	4	(100)	4	(100)	4	(100)	4	(100)	0	(0)	0	(0)
*Enterococcus faecium*	8	8	(100)	8	(100)	8	(100)	8	(100)	0	(0)	0	(0)
*Corynebacterium accolens*	1	1	(100)	1	(100)	1	(100)	1	(100)	0	(0)	0	(0)
*Corynebacterium jeikeium*	1	0	(0)	0	(0)	0	(0)	0	(0)	1	(100)	1	(100)
*Corynebacterium pseudodiphtheriticum*	1	1	(100)	1	(100)	1	(100)	1	(100)	0	(0)	0	(0)
*Corynebacterium simulans*	1	1	(100)	1	(100)	1	(100)	1	(100)	0	(0)	0	(0)
*Corynebacterium striatum*	2	2	(100)	2	(100)	2	(100)	2	(100)	0	(0)	0	(0)
*Turicella otitidis*	1	1	(100)	1	(100)	1	(100)	1	(100)	0	(0)	0	(0)
*Trueperella bernardiae*	1	1	(100)	1	(100)	1	(100)	1	(100)	0	(0)	0	(0)
**Gram-negative bacteria**													
*Escherichia coli*	10	10	(100)	10	(100)	10	(100)	10	(100)	0	(0)	0	(0)
*Acinetobacter* spp.	2	1	(50)	0	(0)	1	(50)	0	(0)	1	(50)	2	(100)
*Enterobacter cloacae* complex^*c*^	2	1	(50)	2	(100)	0	(0)	0	(0)	1	(50)	0	(0)
*Klebsiella aerogenes*	2	2	(100)	2	(100)	2	(100)	2	(100)	0	(0)	0	(0)
*Klebsiella oxytoca*	2	2	(100)	2	(100)	2	(100)	2	(100)	0	(0)	0	(0)
*Klebsiella pneumoniae*	6	5	(83)	6	(100)	5	(83)	6	(100)	1	(17)	0	(0)
*Serratia liquefaciens*	1	1	(100)	1	(100)	1	(100)	1	(100)	0	(0)	0	(0)
*Serratia marcescens*	3	3	(100)	3	(100)	3	(100)	3	(100)	0	(0)	0	(0)
*Morganella morganii*	3	3	(100)	3	(100)	3	(100)	3	(100)	0	(0)	0	(0)
*Proteus mirabilis*	6	6	(100)	6	(100)	6	(100)	6	(100)	0	(0)	0	(0)
*Proteus vulgaris*	1	1	(100)	1	(100)	1	(100)	1	(100)	0	(0)	0	(0)
*Providencia rettgeri*	3	3	(100)	3	(100)	3	(100)	3	(100)	0	(0)	0	(0)
*Providencia stuartii*	1	1	(100)	1	(100)	1	(100)	1	(100)	0	(0)	0	(0)
*Pseudomonas aeruginosa*	8	8	(100)	8	(100)	8	(100)	8	(100)	0	(0)	0	(0)
*Stenotrophomonas maltophilia*	7	7	(100)	7	(100)	7	(100)	7	(100)	0	(0)	0	(0)
*Achromobacter xylosoxidans*	1	1	(100)	1	(100)	0	(0)	0	(0)	0	(0)	0	(0)
*Ochrobactrum anthropi*	1	1	(100)	1	(100)	1	(100)	1	(100)	0	(0)	0	(0)
*Delftia acidovorans*	1	1	(100)	1	(100)	1	(100)	1	(100)	0	(0)	0	(0)
**Anaerobic bacteria**													
*Clostridium perfringens*	5	5	(100)	5	(100)	5	(100)	5	(100)	0	(0)	0	(0)
*Bacteroides fragilis*	3	3	(100)	3	(100)	3	(100)	3	(100)	0	(0)	0	(0)
*Cutibacterium acnes*	5	5	(100)	5	(100)	5	(100)	5	(100)	0	(0)	0	(0)
**Yeast**													
*Candida albicans*	3	3	(100)	3	(100)	3	(100)	3	(100)	0	(0)	0	(0)
*Candida auris*	3	3	(100)	3	(100)	3	(100)	3	(100)	0	(0)	0	(0)
*Candida glabrata*	3	3	(100)	3	(100)	3	(100)	3	(100)	0	(0)	0	(0)
*Cryptococcus neoformans*	5	4	(80)	3	(60)	4	(80)	3	(60)	1	(20)	2	(40)
*Malassezia pachydermatis*	1	1	(100)	1	(100)	1	(100)	1	(100)	0	(0)	0	(0)
Total	154	148	(96)	146	(95)	139	(90)	136	(88)	6	(4)	8	(5)

^*a*^ Both PRIME methods misidentified all *Staphylococcus argenteus* isolates as *Staphylococcus aureus* using KB3.2 database.

^*b*^ ID obtained by sequencing.

^*c*^ *E. xiangfangensis* species level ID was grouped into *E. cloacae* complex per laboratory reporting procedure.

Overall, the Biotyper identified 99% of isolates to the genus level, whereas the PRIME PICKME method resulted in a species-level identification for 139 isolates (90.3%), a genus-level identification for 148 isolates (96.1%), and no identification for 6 isolates (3.9%). With the PRIME Loop method, 136 isolates (88.3%) yielded a species-level identification, 146 isolates (94.8%) a genus-level identification, and 8 isolates (5.2%) were not identified. Eleven isolates were sequenced for discrepant calls between the SOC method (Biotyper) vs PRIME (Table S4). Notably, five isolates that were identified as *S. argenteus,* a member of the *S. aureus* complex (15), by Biotyper and WGS were misidentified as *S. aureus* (species-level confidence) by the PRIME using both the PICKME and Loop methods. Seven of 11 discrepant isolates, including *Staphylococcus argenteus*, are absent from the PRIME v3.2 database. Excluding organisms absent from the database, 95% and 93% of organisms were accurately identified to the species level by the PRIME PICKME and PRIME Loop methods, respectively. After discrepant analyses, 95% and 99% of organisms were accurately identified to the species and genus level, respectively, by Biotyper.

### Identification of isolates from blood cultures: short vs routine incubation

Isolate identification from positive blood cultures (*n* = 300) was evaluated after short (6–8 h) and routine (18–24 h) incubation of subcultured bottles by an HEU (Fig. 1; Fig. S1). Twenty-one of 300 (7%) positive blood cultures were polymicrobial with ≥2 organisms identified, for a total of 322 isolates evaluated. Isolates that failed to identify via either MALDI-TOF MS system or isolates that had discrepant MALDI-TOF MS identifications are listed in Table S5. There were four notable misidentifications: (i) *S. epidermidis* was identified as *Actinomyces timonensis* by Biotyper at short incubation, (ii) *S. cerevisiae* was identified as *Neisseria mucosa/sicca* by PRIME at short incubation when using a loop, (iii) *K. variicola* was called *K. pneumoniae* by PRIME after routine incubation when using a loop, and (iv) Biotyper identified *Corynebacterium hesseae* as *Corynebacterium aurimucosum*.

Overall, the rates of species-level identification for the Biotyper (269, 84%), PRIME PICKME (259, 80%), and PRIME Loop (262, 81%) were similar, and no significant differences were seen between the groups (all *P* values > 0.05, bootstrap resample method) after short incubation from positive blood culture. Genus-level only identifications were obtained for an additional 6 (1.9%), 5 (1.6%), and 4 (1.2%) isolates for the Biotyper, PRIME PICKME, and PRIME Loop, respectively (Table 2). In comparison, after routine incubation, the Biotyper, PRIME PICKME, and PRIME Loop identified 319 (99%), 307 (95%), and 307 (95%) isolates, respectively, to the species level with no significant differences observed between the groups (all *P*-values > 0.05, bootstrap resampling method). After short incubation, yeast had the lowest yield, with only 22%–44% of isolates resulting in an identification, whereas 89%–92% of gram-negative isolates were identified, and 79%–83% of gram-positive isolates were identified. There was no advantage in the use of the PICKME over the Loop for the HEU, as the number of isolates receiving at least a species-level identification was similar after short (PICKME: 82% vs Loop: 83%, *P* = 0.64, McNemar’s) and routine (PICKME: 95% vs Loop: 95%, *P* = 0.68, McNemar’s) incubations.

**TABLE 2:** Comparison of isolate identification after short vs routine incubation of plates from positive blood cultures

Method*^a^*	Biotyper				PRIME PICKME				PRIME Loop			
Incubation	Short		Routine		Short		Routine		Short		Routine	
	No.	(%)	No.	(%)	No.	(%)	No.	(%)	No.	(%)	No.	(%)
Genus ID	275	(85)	319	(99)	264	(82)	307	(95)	266	(83)	307	(95)
Species ID	269	(84)	319	(99)	259	(80)	302	(94)	262	(81)	300	(93)
No ID	47	(15)	3	(1)	58	(18)	15	(5)	56	(17)	15	(5)
# Repeats (*n* = 300 specimens)	34	(11)	6	(2)	53	(18)	18	(6)	53	(18)	17	(5.6)
**Species-level ID categorized by Gram stain morphology**												
Gram-positive (*n* = 230)	191	(83)	229	(100)	181	(79)	21	(94)	184	(80)	215	(93)
Gram-negative (*n* = 83)	76	(92)	82	(99)	74	(89)	77	(93)	74	(89)	76	(92)
Yeast (*n* = 9)	2	(22)	8	(89)	4	(44)	8	(89)	4	(44)	9	(100)
**Time-to-result analysis** *^b^*												
	**Hr**	**(SD)**	**Hr**	**(SD)**	**Hr**	**(SD)**	**Hr**	**(SD)**	**Hr**	**(SD)**	**Hr**	**(SD)**
**Average TTR** *^c^*	0.58	(0.25)			0.45	(0.14)	0.44	(0.34)	0.47	(0.15)	0.44	(0.10)

^*a*^ Results shown are from evaluation by the HEU.

^*b*^ Time-to-result (TTR), the time from setup to the time of the MALDI-TOF MS result, does not include isolates with no identification or second/third organisms in a polymicrobial specimen.

^*c*^ Average TTR was unable to be evaluated for the routine incubation period on Biotyper due to delayed target analysis caused by the daily use of the instrument for routine patient care.

In polymicrobial cultures (*n* = 21), the Biotyper, PRIME PICKME, and PRIME Loop identified 17, 16, and 17 isolates, respectively, to species-level after short incubation with no need for further sub-culture for colony isolation, with the Biotyper identifying one additional organism to genus-level only. These data correspond to an identification (genus- or species-level) of at least one organism in 76%–86% of polymicrobial specimens, with 0 misidentifications.

Attempting identification after short incubation was associated with the need for significantly more MALDI-TOF MS retesting for all methods compared to routine incubation (Biotyper: 34 repeats [short] vs 6 repeats [routine], *P* < 0.0001; PRIME PICKME: 53 repeats [short] vs 18 repeats [routine], *P* < 0.0001; PRIME Loop: 53 repeats [short] vs 17 repeats [routine], *P* < 0.0001; McNemar’s). The Biotyper required significantly fewer repeats than both the PRIME PICKME (34 vs 53 repeats, *P* = 0.01, McNemar’s) and the PRIME Loop (34 vs 53 repeats, *P* = 0.01) at short incubations.

The time-to-result (TTR)—defined as the time from setup to the time of the MALDI-TOF MS result—was 0.58 h, 0.45 h, and 0.47 h for the Biotyper, PRIME PICKME, and PRIME Loop, respectively, for the short incubation (Table 2). The PRIME PICKME and PRIME Loop TTR were both significantly shorter compared with the Biotyper (PICKME: −7.8 min, *P* < 0.0001; Loop: −6.6 min, *P* < 0.0001; one-way ANOVA/Tukey’s).

### Identification of isolates from blood cultures: HEU vs LEU

A subset of 50 blood culture specimens was evaluated by both an HEU and an LEU after short and routine incubation. Three specimens were polymicrobial, for a total of 53 isolates. At short incubation, the performance for final identification was similar between the HEU and LEU for all methods with no significant difference in rates of species-level identification for Biotyper (89% vs 85%; *P* = 0.79, Fisher’s exact), PRIME PICKME (89% vs 79%, *P* = 0.31), or PRIME Loop (91% vs 83%, *P* = 0.42), and no significant difference in rates of species-level identification for the LEU using PRIME PICKME in comparison to PRIME Loop (79% vs 83%; *P* = 0.75, McNemar’s) (Table 3). Notably, species-level identification rates for the LEU were 20% lower for gram-negative isolates using the PRIME Loop compared with PRIME PICKME and Biotyper (Table 3). At short incubation, there was no difference in the number of repeats needed for the Biotyper between the HEU and LEU (*n* = 3 for both), whereas the HEU needed fewer repeats compared with the LEU for the PRIME PICKME (4 vs 10, *P* = 0.15, Fisher’s exact) and PRIME Loop methods (5 vs 13, *P* = 0.07), although this was not statistically significant. At routine incubation, the performance for final identification was similar between the HEU and LEU for all methods, although the LEU required more repeat MALDI-TOF MS attempts using the PRIME Loop compared with the HEU (5 repeats vs 0, *P* = 0.02, Fisher’s exact, Table S6).

**TABLE 3:** Comparison of isolate identification for HEU vs LEU after short incubation of plates from positive blood cultures

Method	Biotyper				PRIME PICKME				PRIME Loop			
User*^a^*	HEU		LEU		HEU		LEU		HEU		LEU	
	No.	(%)	No.	(%)	No.	(%)	No.	(%)	No.	(%)	No.	(%)
Genus ID	48	(91)	47	(89)	47	(89)	42	(79)	48	(91)	44	(83)
Species ID	47	(89)	45	(85)	47	(89)	42	(79)	48	(91)	44	(83)
No ID	5	(9.4)	6	(11)	6	(11)	11	(21)	5	(9.4)	9	(17)
# Repeats (*n* = 50 specimens)	4	(8)	4	(8)	4	(8)	10	(20)	5	(10)	13	(26)
**Species-level ID categorized by Gram-stain morphology**												
Gram-positive (*n* = 36)	32	(89)	30	(83)	32	(89)	27	(75)	33	(92)	31	(86)
Gram-negative (*n* = 15)	15	(100)	15	(100)	15	(100)	15	(100)	15	(100)	12	(80)
Yeast (*n* = 2)	0	(0)	0	(0)	0	(0)	0	(0)	0	(0)	1	(50)
**Time-to-result (TTR) analysis** *^b^*												
	**Hr**	**(SD)**	**Hr**	**(SD)**	**Hr**	**(SD)**	**Hr**	**(SD)**	**Hr**	**(SD)**	**Hr**	**(SD)**
**Average TTR**	0.65	(0.18)	0.57	(0.18)	0.57	(0.19)	0.52	(0.15)	0.54	(0.17)	0.52	(0.21)

^*a*^ High-experience user (HEU), >1 year of MALDI-TOF MS experience. Low-experience user (LEU), <1 month of MALDI-TOF MS experience.

^*b*^ Time to result (TTR), the time from setup to the time of the MALDI-TOF MS result.

### Timing evaluation for set up and analysis of a single target

**Fig 3:**
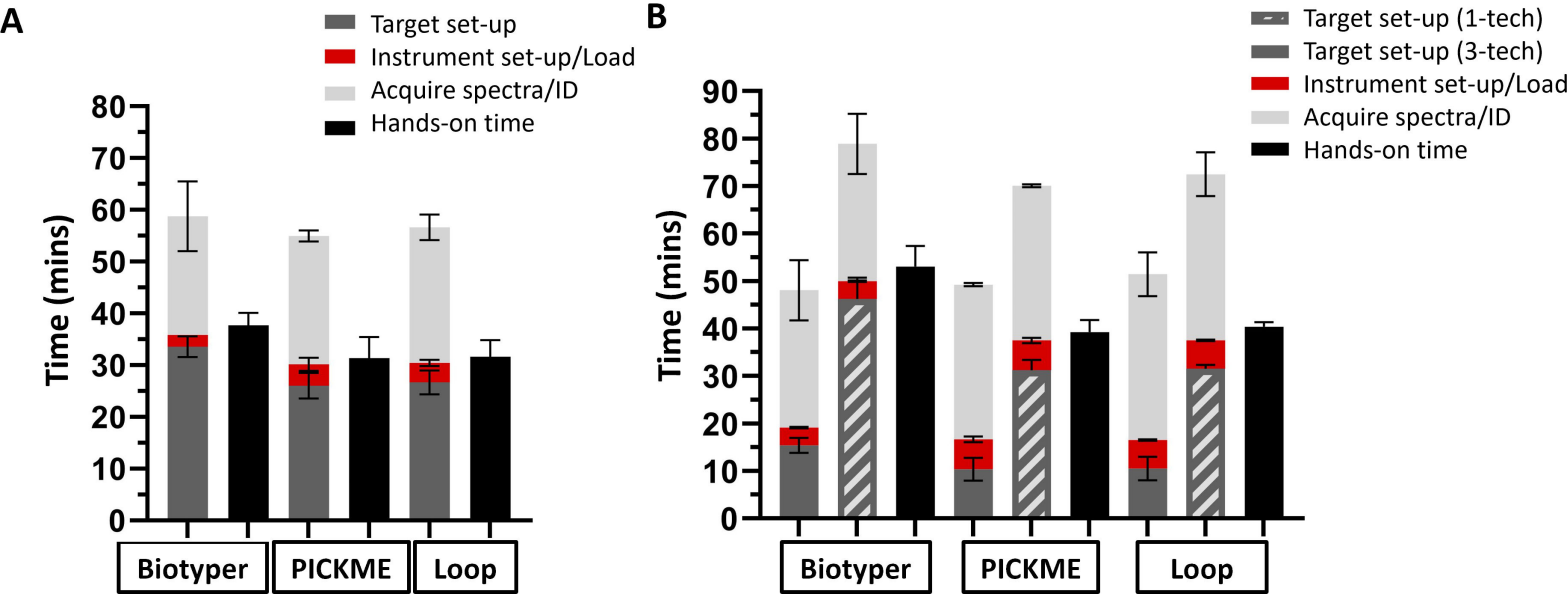
(**A**) Single-target workflow time study results. Experiments were performed in duplicate by both an HEU and an LEU, for a total of four targets (four replicates) per method. The following steps were measured: “Target set-up” (dark gray bars): time it takes to add barcodes to a target sheet to label spots, spot 48 isolates on the target, and add and dry formic acid (when applicable) and matrix; “Instrument set-up/load” (red bars): time for instrument setup, sample layout setup via barcode scanning, and target loading onto the instrument; and “Acquire spectra/ID” (light gray bars): time for the vacuum seal to establish after the target is inserted, calibration, data acquisition, display of organism identifications, and result review. “Hands-on time” (black bars) included any step where the user was actively engaged and included target and instrument set-up and loading [+calibration step for Biotyper only], result review, and target unloading. (**B**) Multi-target workflow time study results; 48 isolates were evenly distributed across three individual targets for each method. Experiments were performed in duplicate by both an HEU and an LEU, for a total of 12 targets (four replicates) per method. The components measured for the steps listed above. “Total process completion time” and “hands-on time” were the same as for the single-target workflow. However, since the multi-target workflow could be accomplished via two main pathways depending on a laboratory’s staffing and culture volume, the “Target set-up” step was simulated in two different ways. The three-tech workflow (dark gray bars) was timed to simulate three technologists simultaneously setting up one target with 16 spots. In the one-tech workflow (striped bars), simulating a laboratory with a dedicated MALDI technologist, one technologist sequentially set up three targets, each with 16 spots. Error bars represent the standard deviation.

Timing of steps for the single-target workflow for the Biotyper, PRIME PICKME, and PRIME Loop methods were compared (Fig. 2). Overall, the total process completion time was similar for all methods (Biotyper: 59.37 min, PRIME PICKME: 55.47 min, PRIME Loop: 57.15 min; *P* = 0.63, one-way ANOVA). Although, depending on the instrument and method, different steps took longer (Fig. 3A). First, the time to set up 48 spots was different between instruments (Biotyper: 33.55 min, PRIME PICKME: 26.05 min, PRIME Loop: 26.68 min), with the PRIME PICKME (−7.5 min, *P* = 0.0030, one-way ANOVA/Tukey’s) and PRIME Loop (−6.87 min, *P* = 0.0052, one-way ANOVA/Tukey’s) being faster than the Biotyper. Next, the time to set up the instrument and load the target was different (Biotyper: 2.30 min, PRIME PICKME: 4.13 min, PRIME Loop: 3.75 min), with the PRIME PICKME taking longer than Biotyper (+1.83 min, *P* = 0.03, one-way ANOVA/Tukey’s), and the PRIME Loop taking longer than Biotyper (+1.45 min, *P* = 0.08, one-way ANOVA/Tukey’s), although not statistically significant. There was no difference in instrument run time (acquire spectra and obtain ID) between the methods (Biotyper: 22.92 min, PRIME PICKME: 24.80 min, PRIME Loop: 26.20 min, *P* = 0.56, one-way ANOVA). Average overall hands-on time was significantly longer for the Biotyper (53.0 min [std dev 4.4]) in comparison with the PRIME PICKME (39.3 min [2.3]; *P* < 0.001, one-way ANOVA/Tukey’s) and PRIME Loop (40.4 min [1.0], *P* < 0.001).

### Comparison of workflow for setup and analysis of multiple targets

The time to set up targets for the multi-target workflow was examined in two ways, simulating either three technologists spotting one target with 16 isolates each to mimic a workflow where targets are set up by multiple people working different benches (three-tech workflow), or one technologist sequentially spotting three targets with 16 isolates each to mimic the workflow of a clinical lab with one MALDI-TOF MS technologist processing targets for all benches (one-tech workflow). The time to set up 16 spots (three-tech workflow) was longer for the Biotyper (15.40 min) vs PRIME PICKME (10.40 min, *P* = 0.024, one-way ANOVA/ Tukey’s) and PRIME Loop (10.52 min, *P* = 0.028) methods, with no difference between the PRIME PICKME and PRIME Loop (*P* = 1.0). The time to set up 48 spots (one-tech workflow) was also longer for the Biotyper (46.22 min) vs PRIME PICKME (31.23 min, *P* = 0.0001, one-way ANOVA/Tukey’s) and PRIME Loop (31.55 min, *P* = 0.0002). Other steps in the multi-target workflow were not considered to be impacted by the number of technologists working simultaneously, so were only evaluated using one version of the metric. The time to set up instrument and load targets was faster for the Biotyper (3.77 min) vs PRIME PICKME (6.27 min, *P* < 0.0001, one-way ANOVA/Tukey’s) and PRIME Loop (6.00 min, *P* < 0.0001) methods. There was no difference in instrument run time between the three methods (*P* = 0.22, one-way ANOVA).

Total process completion time was impacted by the number of technologists working simultaneously and was shorter for the three-tech vs one-tech workflow for each method (PRIME PICKME: 50.55 min vs 71.37 min, *P* < 0.0001; PRIME Loop: 52.95 min vs 74 min, *P* = 0.0004; unpaired *t*-test). For the three-tech workflow, the total process completion time was not different between the three methods (*P* = 0.37, one-way ANOVA). However, for the one-tech workflow, total process completion time was different between methods (Biotyper: 79.48 min, PRIME PICKME: 71.37 min, PRIME Loop: 74.0 min, *P* = 0.04, one-way ANOVA), with the Biotyper taking longer than both PRIME PICKME (+8.11 min, *P* = 0.04, one-way ANOVA/Tukey’s) and PRIME Loop (+5.48 min, *P* = 0.61), although not statistically significant. Total hands-on time was not considered to be impacted by the number of technologists working simultaneously. Total hands-on time was greater for the Biotyper (53.03 min) vs PRIME PICKME (39.25 min, *P* = 0.0003, one-way ANOVA/Tukey’s) and PRIME Loop (40.35 min, *P* = 0.0005) methods.

### VITEK MS PRIME urgent slide analysis

Time savings from using the urgent slide analysis feature on the PRIME was evaluated in a three-target experiment (Fig. 2), where a single isolate spot on the third target was designated as urgent (i.e., target 3/spot 1 will be prioritized over target 2). The average time savings to identification from using this feature (which skipped one slide with one acquisition group in this experiment) was 11.15 min (+/− 0.38 min SD).

## DISCUSSION

The results of this study highlight the VITEK MS PRIME’s performance in identifying a wide range of bacterial and yeast isolates and its efficiency in clinical workflows. The PRIME demonstrated high accuracy for species-level identifications of database-included isolates and performed comparably with the Biotyper across various experimental conditions, including identifying over 80% of blood culture isolates after short incubation on solid media. PRIME workflows, particularly in high-throughput settings, also offered significant time savings in hands-on processing and urgent identifications, emphasizing its potential to enhance laboratory efficiency.

Faster identification of organisms from a positive blood culture can facilitate patient management and provide necessary organism identification for the interpretation of rapid phenotypic antimicrobial susceptibility results. Thus, we assessed the performance of Biotyper and PRIME for the accurate identification of isolates after short incubation. We found that early microorganism identification after short incubation on solid media enables reliable results without additional hands-on time or extraction, allowing species-level identification from typically monomicrobial specimens, such as blood cultures, within a single 8 h shift. Our study demonstrated that species-level identification was achievable for most isolates after short incubation, regardless of user experience or instrument used, even in polymicrobial cultures. It is notable that individual colonies were often very pinpoint or not present after a short incubation. In these cases, identification was attempted on scum growth, and additional organisms became more apparent after routine incubation. Because organisms may be missed at early incubation stages in polymicrobial cultures, routine incubation of plates is still necessary to ensure that all organisms are identified. Misidentifications at the genus level were rare, with only one instance each for PRIME (with loop) and Biotyper out of 300 specimens analyzed. However, short-term incubation increased the need for repeat MALDI-TOF MS attempts for all methods after a first failed attempt, which may extend the time-to-result (TTR) for some isolates. Identification success was the highest for gram-negative (89%–92%) and gram-positive bacteria (79%–83%), whereas yeast identification was less successful (22%–44%) due to slower growth and smaller colony sizes (Fig. S1). Of note, the application of Bruker recommended cutoffs for species-level identification (>2.0) would have resulted in lower overall rates of species-level identification of 73% after short incubation, and 95% with routine incubation, in our data set. Somewhat similar success rates were achieved in a prior study that analyzed the identification of microcolonies after 4 h of growth from 218 positive blood cultures, where the Biotyper identified 55% of isolates to the species level and PRIME identified 70.2% (16).

Organism misidentification poses a potential risk to patients. In this study, most misidentifications for the PRIME occurred when using the loop (*S. cerevisiae, K. variicola, S. anginosus* subsp. *whileyi, S. borealis,* and *S. argenteus*). Notably, only one of these cultures was polymicrobic. Misidentifications with the PRIME PICKME occurred with organisms not available in the v3.2 library (five *S. argenteus* and one *S. borealis*). Some of the misidentifications could be caught by simple phenotypic tests, such as gram stain morphology, whereas others like *S. argenteus* vs *S. aureus* would be missed, although the clinical impact of this misidentification would likely be minimal. Both misidentified and non-identified organism rates will likely improve with new iterations of the database as new organisms, including *S. argenteus* and *S. petrasii*, have been added to the VITEK MS KB 3.3 library. Five misidentifications were observed with the Biotyper (*Corynebacterium hesseae, Corynebacterium macclintockiae, Enterobacter roggenkampii, Staphylococcus epidermidis,* and *Streptococcus dysgalactiae* subsp*. equisimilis)*, for which both *Corynebacterium* species are not available in the Biotyper library used in this study.

Given some of the key differences between the PRIME and Biotyper systems in target design and analysis (Table S1), we assessed laboratory workflows for the different methods. Biotyper and PRIME offer time-savings advantages at different steps. PRIME requires less hands on time, which can be an advantage for labs that are short-staffed. The Biotyper instrument has faster setup/operation, with greater flexibility for the number of spots in a run and reuse of targets, which can be an advantage for labs that use fewer spots at a time per target. Target setup and hands on time were greater for Biotyper workflows due to the longer spotting and formic acid/matrix drying time noted in our study. However, the additional formic acid step and drying time may not be standard of care for all laboratories that use the Biotyper and may be alleviated by use of the MBT FAST shuttle to reduce dry time or use of formic acid only for yeast or organisms that fail to achieve initial identification.

Staffing shortages and training challenges are becoming more prevalent in clinical laboratories (17), which has contributed to overall lower-experienced technologists in the clinical laboratory workforce. To this end, we compared the performance of two spotting methods for users at different experience levels. For PRIME, the PICKME method yielded comparable results to the Loop but yielded correct identifications for two database-included organisms that were misidentified with the Loop. Additionally, the use of the PICKME nib increased species-level identifications (100%) for gram-negative organisms for the LEU in comparison to standard set-up using the Loop (80%), likely due to the more uniform application of larger gram-negative colonies to the target through the use of the PICKME nib.

With staffing shortages and the expanding capabilities of total laboratory automation systems, workflow considerations are increasingly important to laboratories. Based on this study, both systems provided rapid and accurate identification of isolates when performed by MALDI-TOF MS users with varying levels of experience. The PRIME offered faster target setup and time-to-results, whereas the Biotyper identified more organisms overall, required fewer repeats, and had a faster instrument setup time. In contrast, Thelen et al. found that Biotyper Sirius had shorter hands on time (by 3–6 min) and faster instrument measurement speed compared with PRIME, whereas target preparation did not differ between the two systems (18). The difference is likely due to our laboratory practice of using formic acid on all isolates for Biotyper.

Strengths of this study include the use of diverse isolates, including those from positive blood cultures, analysis of different user experience levels, and different workflow approaches (PICKME vs Loop). Limitations of the study include the lack of anaerobic data for blood culture isolates and the generalizability of our standard of care Biotyper method, which may not be reflective of other clinical labs.

In conclusion, this study demonstrates the robust performance of the VITEK MS PRIME in identifying a broad range of bacterial and yeast isolates, with a possibility for enhanced efficiency in clinical laboratory workflows. By enabling reliable species-level identification after short incubation, the PRIME shows potential to support faster diagnostic turnaround times, especially in high-throughput and urgent settings. Although both PRIME and Biotyper systems offered comparable accuracy, each exhibited distinct workflow advantages, underscoring the importance of aligning technological capabilities with laboratory needs. The integration of such advanced diagnostic tools into clinical workflows can optimize resource utilization, address staffing challenges, and improve laboratory throughput. Further studies incorporating additional organism types will help refine the application of these systems and maximize their impact on patient care.

## SUPPLEMENTAL MATERIAL

Supplemental tables and figure jcm.00211-25-s0001.pdf Tables S1 to S6 and Fig. S1.

ASM does not own the copyrights to Supplemental Material that may be linked to, or accessed through, an article. The authors have granted ASM a non-exclusive, world-wide license to publish the Supplemental Material files. Please contact the corresponding author directly for reuse.
