# Supplementary material for: Performance and workflow comparison of the VITEK MS PRIME and Bruker Biotyper MALDI-TOF MS systems
Source: J Clin Microbiol. 2025 Jun 20;63(8):e00211-25. doi: 10.1128/jcm.00211-25 (PMC12345239; doi:10.1128/jcm.00211-25)
Supplement: Supplemental tables and figure — Tables S1 to S6 and Fig. S1. [file jcm.00211-25-s0001.pdf]

SUPPLEMENTARY TABLES AND FIGURES

Supplementary Table 1. Comparison of Bruker Biotyper and VITEK MS PRIME features.

Feature	MALDI Biotyper	VITEK MS PRIME
**Instrument capacity; Target spot capacity**	• 1 target<br>• 96 spots	• 16 targets<br>• 1 target has 48 spots, separated into 3 acquisition groups with 16 spots each
**Target loading**	Single target	• Can load additional targets while instrument is running<br>• Ability to prioritize an "urgent" target
**Type of target(s)**	• Disposable<br>• Reusable	• Disposable
**Target spotting tools**	Flexible. Typically:<br>• Loops<br>• Toothpicks	• Loop<br>• PICKME pen
**Formic acid step**	Used on all organisms (standard of care/routine workflow in our laboratory)	Used on yeast only
**Layout setup (barcode scanning of patient labels)**	Instrument automatically proceeds to next spot during scanning	Instrument requires a keyboard touch between spots to proceed to the next
**Target acquisition groups**	Full target can be acquired as 1 (or more) group (based on 1 calibration)	Full target is acquired as 3 groups (based on 3 calibrations)
**Calibration timing**	Occurs at beginning of run	Occurs at beginning & end of run
**Result/organism identification viewing**	As available; displayed as acquisition of spots proceeds	Batched; displayed at the end of a group's acquisition
**Repeat acquisition of spots**	Can be re-shot/re-acquired after use (including the control)	Spots cannot be re-shot/re-acquired after use
**Reuse of targets with leftover/empty spots**	Can reuse targets with empty spots	Can only reuse targets if ≥1 unused acquisition group(s)

Supplementary Table 2. Isolates (n=322) from positive blood cultures (n=300).

Organism	No. of Isolates
**Gram-positive bacteria**	**230**
Aerococcus species	1
Aerococcus viridans	1
Bacillus altitudinis/pumilus	1
Bacillus cereus group	2
Clostridium tertium	1
Corynebacterium aurimucosum	1
Corynebacterium coyleae	1
Corynebacterium hesseae	1
Corynebacterium species	1
Corynebacterium striatum	3
Enterococcus faecalis	16
Enterococcus faecium	8
Microbacterium species	1
Rothia dentocariosa	1
Staphylococcus aureus	74
Staphylococcus capitis	10
Staphylococcus cohnii	1
Staphylococcus epidermidis	53
Staphylococcus borealis	1
Staphylococcus haemolyticus	4
Staphylococcus hominis	15
Staphylococcus petrasii	1
Staphylococcus pettenkoferi	3
Staphylococcus simulans	2
Streptococcus agalactiae	5
Streptococcus anginosus	5
Streptococcus constellatus	1
Streptococcus dysgalactiae	2
Streptococcus gallolyticus	1
Streptococcus mitis/Streptococcus oralis	3
Streptococcus pneumoniae	5
Streptococcus pyogenes	2
Streptococcus salivarius group	3
**Gram-negative bacteria**	**83**
Acinetobacter pittii	1
Acinetobacter variabilis	1
Citrobacter freundii	1
Citrobacter koseri	1

Enterobacter cloacae complex	5
Escherichia coli	38
Herbaspirillum huttiense	1
Klebsiella aerogenes	2
Klebsiella oxytoca	2
Klebsiella pneumoniae	12
Klebsiella variicola	1
Mixta calida	1
Paracoccus yeei	1
Proteus mirabilis	6
Proteus vulgaris	1
Pseudomonas aeruginosa	4
Pseudomonas putida group	1
Salmonella species	2
Serratia marcescens	2
**Yeast**	**9**
Candida albicans	1
Candida glabrata	3
Candida krusei	1
Candida parapsilosis	1
Candida tropicalis	2
Saccharomyces cerevisiae	1

Supplementary Table 3. Isolates (n=48) included in the workflow analysis.

Organism	# of strains tested
Candida albicans	4
Candida glabrata	4
Enterococcus faecalis	4
Escherichia coli	4
Klebsiella pneumoniae	4
Proteus mirabilis	4
Pseudomonas aeruginosa	4
Staphylococcus aureus	4
Staphylococcus epidermidis	4
Stenotrophomonas maltophilia	4
Streptococcus mitis group	4
Streptococcus agalactiae	4

Supplementary Table 4. Discrepant analysis for the performance evaluation.

BIOTYPER ID	ID Level	PRIME PICKME ID	ID Level	PRIME LOOP ID	ID Level	Final Reference ID (WGS or 16S-based)
No ID (n=1)	NA	*Staphylococcus cohnii* ssp. *urealyticus*	Species	*Staphylococcus cohnii* ssp. *urealyticus*	Species	*Staphylococcus ureilyticus*
Corynebacterium jeikeium (n=1)	Species	No ID	NA	No ID	NA	*Corynebacterium macclintockiae*
Cryptococcus neoformans (n=1)	Species	No ID	NA	No ID	NA	*Cryptococcus neoformans*
Staphylococcus pettenkoferi (n=1)	Species	No ID	NA	No ID	NA	*Staphylococcus pettenkoferi*
**Not in VITEK MS PRIME Library KB 3.2**						
Acinetobacter variabilis (n=1)	Genus	No ID	NA	No ID	NA	*Acinetobacter variabilis*
Staphyloccocus argenteus (n=5)	Species	*Staphylococcus aureus*	Species	*Staphylococcus aureus*	Species	*Staphylococcus argenteus*
Streptococcus minor (n=1)	Species	No ID	NA	No ID	NA	*Streptococcus minor*

"ID Level" refers to whether the method yielded a genus- or species-level identification.

Supplementary Table 5. Discrepant analysis for positive blood culture isolates.

BIOTYPER ID	ID Level	PRIME PICKME ID	ID Level	PRIME LOOP ID	ID Level	Final Reference ID (WGS or 16S-based)
No ID (n=1)	NA	No ID	NA	No ID	NA	*Microbacterium* sp.
No ID (n=1)	NA	No ID	NA	No ID	NA	*Paracoccus yeei*
No ID (n=1)	NA	*Candida glabrata*	Species	*Candida glabrata*	Species	*Candida glabrata (Nakaseomyces glabratus)*
No ID (n=1)	NA	*Saccharomyces cerevisiae*	Species	*Neisseria mucosa/sicca* (short[a])	Species	*Saccharomyces cerevisiae*
Aerococcus viridans (n=1)	Species	No ID	NA	No ID	NA	*Aerococcus* sp.
Actinomyces timonensis (n=1)	Species	*Staphylococcus epidermidis*	Species	*Staphylococcus epidermidis*	NA	*Staphylococcus epidermidis*
Corynebacterium aurimucosum (n=1)	Species	No ID	NA	No ID	NA	*Corynebacterium* sp.
Corynebacterium aurimucosum (n=1)	Species	No ID	NA	No ID	NA	*Corynebacterium hesseae*
Corynebacterium coyleae (n=1*)*	Species	No ID	NA	No ID	NA	*Corynebacterium coyleae*
Klebsiella variicola (n=1)	Species	*Klebsiella variicola*	Species	*Klebsiella pneumoniae* (routine)	Species	*Klebsiella variicola*
Staphylococcus borealis/ Staphylococcus haemolyticus (n=2)	Species	*Staphylococcus haemolyticus*	Species	*Staphylococcus haemolyticus*	Species	*Staphylococcus haemolyticus*
Staphylococcus pettenkoferi (n=1)	Species	No ID	NA	No ID	NA	*Staphylococcus pettenkoferi*
Streptococcus anginosus (n=2)	Species	*Streptococcus anginosus*	Species	*Streptococcus constellatus* (routine, n=1) (short, n=1)	Species	*Streptococcus anginosus* ssp. *whileyi*

BIOTYPER ID	ID Level	PRIME PICKME ID	ID Level	PRIME LOOP ID	ID Level	Final Reference ID (WGS or 16S-based)
Streptococcus canis (n=1)	Species	*Streptococcus dysgalactiae* ssp. *dysgalactiae*/ *Streptococcus dysgalactiae* ssp. *equisimilis*	Genus	*Streptococcus dysgalactiae* ssp. *dysgalactiae*/ *Streptococcus dysgalactiae* ssp. *equisimilis*	Genus	*Streptococcus dysgalactiae* ssp. *equisimilis*
**Not in VITEK MS PRIME Library KB 3.2**						
Acinetobacter variabilis (n=1)	Species	No ID	NA	No ID	NA	*Acinetobacter variabilis*
Enterobacter bugandensis (n=1)	Species	No ID	NA	No ID	NA	*Enterobacter roggenkampii*
Mixta calida (n=1)	Species	No ID	NA	No ID	NA	*Mixta calida*
Staphylococcus borealis (n=1)	Species	*Staphylococcus haemolyticus* (both)	Species	*Staphylococcus haemolyticus* (both)	Species	*Staphylococcus borealis*
Staphylococcus petrasii (n=1)	Species	No ID	NA	No ID	NA	*Staphylococcus petrasii*

"ID Level" refers to whether the method gave a genus- or species-level identification.

"No ID" is listed if both routine and short incubations gave no ID. The condition which yielded the discrepant identification is listed (short and/or routine incubation).

[a]Organism identification obtained from early growth in polymicrobial culture.

Supplementary Table 6. Comparison of HEU and LEU MALDI-TOF MS results from routine incubation of positive blood cultures.

Method	Biotyper				PRIME PICK ME				PRIME Loop			
User[a]	HEU		LEU		HEU		LEU		HEU		LEU	
	No.	(%)	No.	(%)	No.	(%)	No.	(%)	No.	(%)	No.	(%)
Genus ID	53	(100)	52	(98)	53	(100)	52	(98)	53	(100)	53	(100)
Species ID	53	(100)	51	(96)	53	(100)	52	(98)	53	(100)	53	(100)
No ID	0	(0)	1	(1.9)	0	(0)	1	(1.9)	0	(0)	0	(0)
# Repeats	2	(4)	2	(4)	0	(0)	2	(4)	0	(0)	5	(10)
**Species level ID by Gram stain morphology**												
Gram-positive (n=36)	36	(100)	36	(100)	36	(100)	35	(97)	36	(100)	36	(100)
Gram-negative (n=15)	15	(100)	14	(93)	15	(100)	15	(100)	15	(100)	15	(100)
Yeast (n=2)	2	(100)	1	(50)	2	(100)	2	(100)	2	(100)	2	(100)
**Time-to-result (TTR) analysis[b]**												
	**Hr**	**(SD)**	**Hr**	**(SD)**	**Hr**	**(SD)**	**Hr**	**(SD)**	**Hr**	**(SD)**	**Hr**	**(SD)**
Average TTR[c]					0.63	(0.76)	0.51	(0.13)	0.48	(0.15)	0.48	(0.14)

[a]High-experience user (HEU), >1 year of MALDI-TOF MS experience. Low-experience user (LEU), <1 month of MALDI-TOF MS experience.

[b]Time to result (TTR), time from setup to the time of the MALDI-TOF MS result.

[c]Average TTR was unable to be evaluated for the routine incubation period on Biotyper due to delayed target analysis caused by daily use of the instrument for routine patient care.

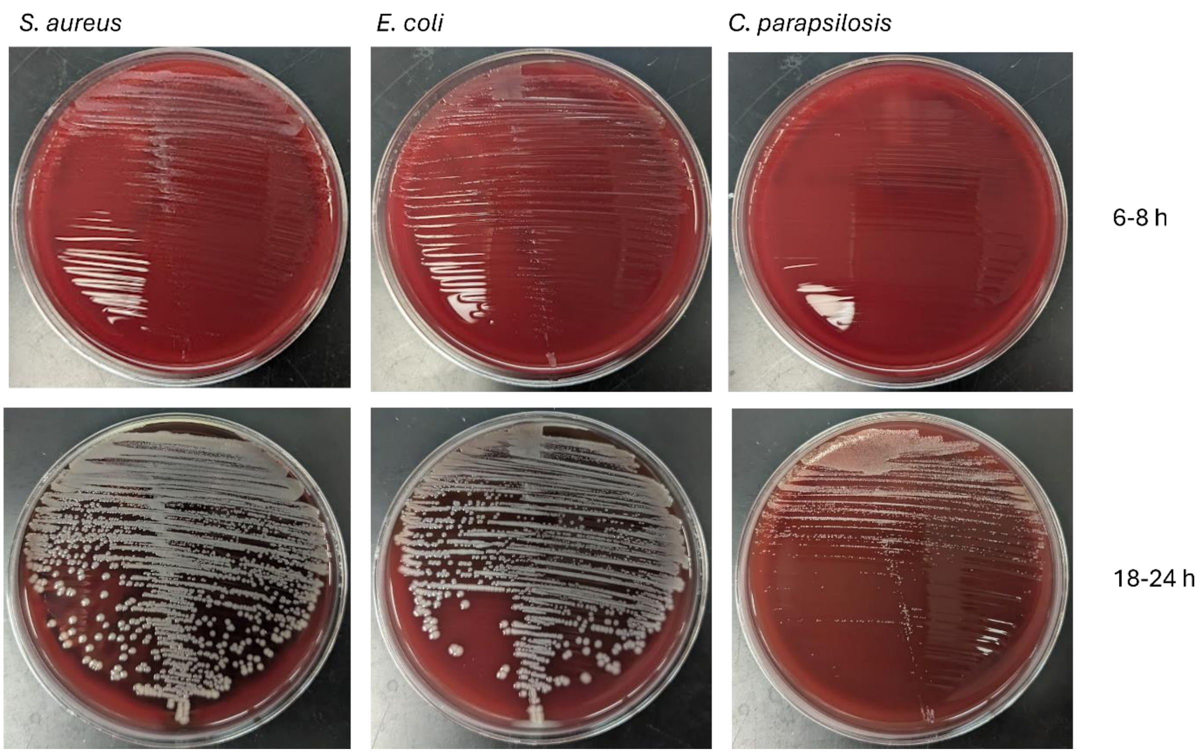

Supplementary Figure 1. Growth of organisms from positive blood cultures at short (6-8 h) and routine (18-24 h) incubations. Positive blood culture bottles were subcultured to blood agar plates and incubated for 6-8 h and 18-24 h at 35°C in 5% CO_2. Examples of growth of Gram-positive bacteria (*S. aureus*), Gram-negative bacteria (*E. coli*), and yeast (*C. parapsilosis*) are shown.
